# The machine learning–powered BirdNET App reduces barriers to global bird research by enabling citizen science participation

**DOI:** 10.1371/journal.pbio.3001670

**Published:** 2022-06-28

**Authors:** Connor M. Wood, Stefan Kahl, Ashakur Rahaman, Holger Klinck

**Affiliations:** K. Lisa Yang Center for Conservation Bioacoustics, Cornell Lab of Ornithology, Cornell University, Ithaca, New York, United States of America

## Abstract

Involving the general public in research through free bird sound identification apps such as BirdNET can generate tens of millions of bird observations globally, helping citizen science to power avian ecology

## Teaser

The BirdNET App, a free bird sound identification app for Android and iOS that includes over 3,000 bird species, reduces barriers to citizen science while generating tens of millions of bird observations globally that can be used to replicate known patterns in avian ecology.

The ubiquity of smartphones combined with the power of new machine learning algorithms presents an opportunity to promote positive interactions between humans and birds and thus create new possibilities for avian research. We present the BirdNET App, a free program capable of identifying over 3,000 bird species by sound alone. The BirdNET App allows users to record audio on a smartphone and transmit that audio and anonymized metadata to the BirdNET server, and a bird species identification is provided with a qualitative confidence score. The raw audio, quantitative confidence score, and metadata (date, time, and location) are saved on the server for subsequent research usage; all observations are anonymized, and no user data are stored. This analytical workflow was inspired in part by the Pl@ntNet app, which combines crowdsourcing and machine learning to engage users in plant identification [1]. The BirdNET App relies on 2 components: the species identification algorithm and the user interface.

**Fig 1:**
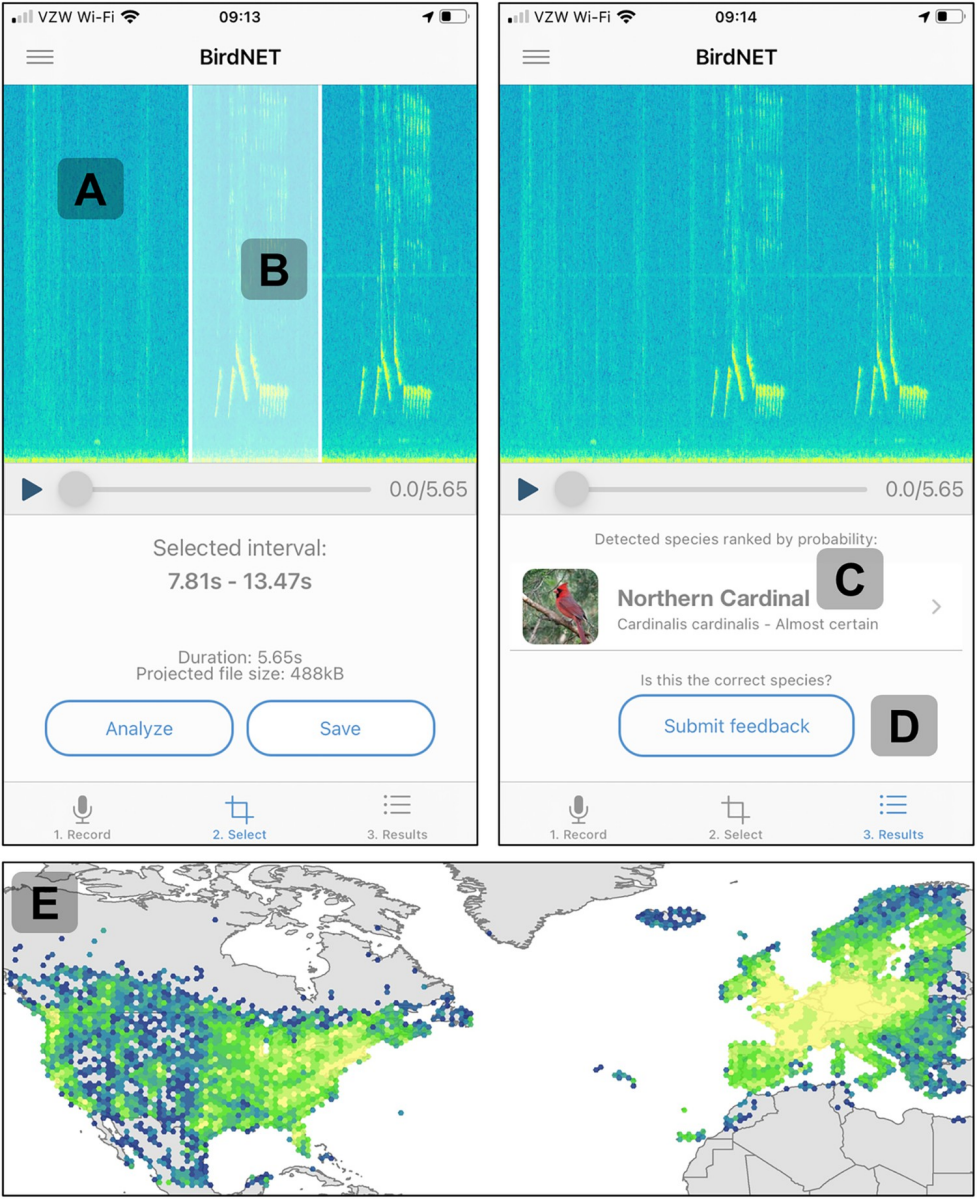
Images of the app user interface (iOS version, American English) and the distribution of submissions. A spectrogram **(A)** visualizes environmental sounds in real time by flowing right to left and is paused when the user selects a snippet of sound for analysis **(B)**. After that snippet is analyzed by the BirdNET server, a qualitative species identification is provided **(C)**, and the user has the option of indicating whether that identification is correct **(D)**. BirdNET App users made 31 million submissions in 2020, and even after stringent quality filters were applied, 5.8 million observations remained and achieved near-total coverage of North America and Europe **(E)**. *Image credit*: *C*. *Wood; base maps were provided by the University of Minnesota* (https://conservancy.umn.edu/handle/11299/227302).

Briefly, the BirdNET algorithm is a deep convolutional neural network. The 2018 to 2020 version could identify 984 North American and European bird species; an early 2021 update enabled it to identify over 3,000 species from around the world. For a full technical description of the algorithm, see [2]. The interface was built around a real-time spectrogram from which users actively select snippets of sound for analysis, a feedback button allowing users to indicate whether a prediction was correct or not, and a basic data portal allowing users to view their own observations and commonly observed species in their area (Fig 1). The app interface supports 13 languages, with species names translated into an additional 12 languages. Our underlying philosophy was that consistently high prediction accuracy with global coverage and a simple, rewarding user interface would help us achieve the sustained use that would be necessary for research applications and most enjoyable for users.

To test whether the app could indeed remove barriers to citizen science—something that is often suggested but rarely tested [3]—we compared usage of the BirdNET App to that of eBird. Both programs enable citizen scientists to submit bird observations for research purposes, but eBird, a pioneering project initiated in 2003, is designed for intermediate/expert birders who can submit checklists of birds they have identified, while the BirdNET app can be used by beginners who have no knowledge of birds. In 2020, the BirdNET App engaged >1.1 million participants compared to 317,792 eBird participants (eBird: An online database of bird distribution and abundance. Ithaca, New York: Cornell Lab of Ornithology; 2021). This difference in participation does not imply that one is “better” than the other; instead, it reflects conscious design choices. By catering to a smaller pool of more advanced users, eBird can generate high-quality data (e.g., abundance and nondetections). By removing the need for preexisting bird identification skills and specialized equipment like binoculars, the BirdNET App vastly expands the number of potential citizen science participants, although it generates more simplistic presence-only data. These paradigms are complementary, and, ideally, synergistic, with an exchange of data and participants leading to improved research tools and conservation outcomes.

**Fig 2:**
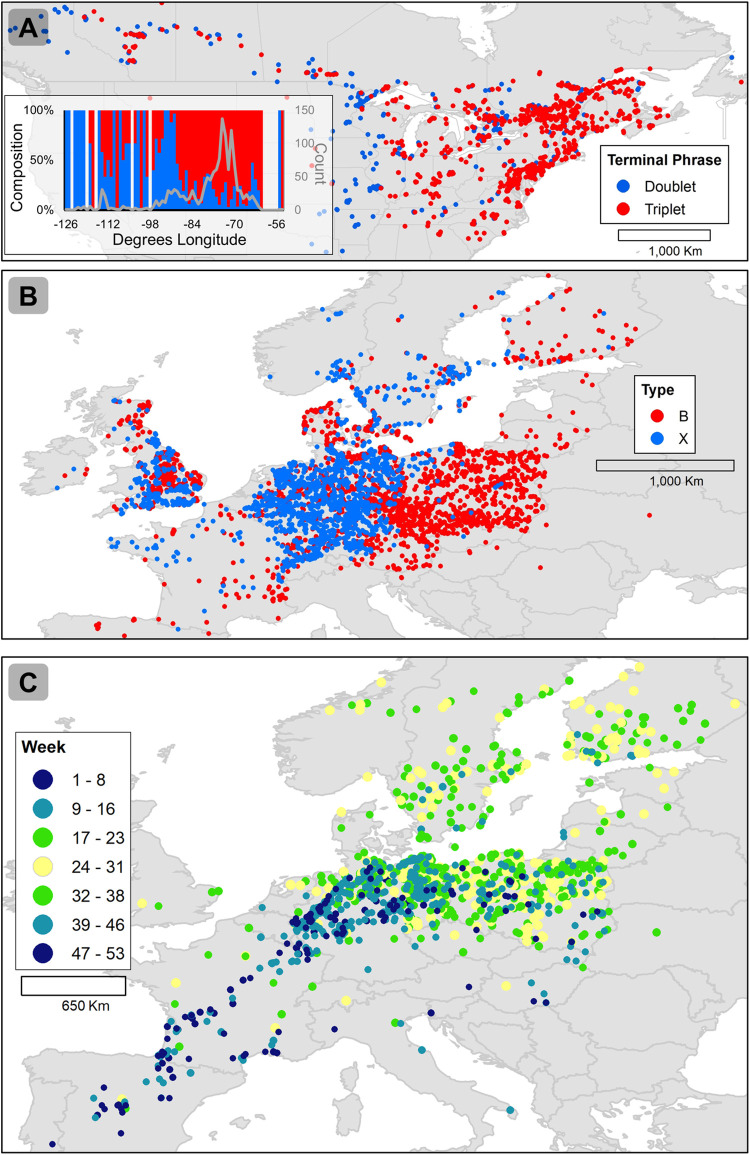
Results from BirdNET App data case studies. BirdNET App submissions of White-throated Sparrow **(A)** and Yellowhammer **(B)** songs were manually sorted into distinct dialects, which reflected the geographically distinct dialects reported by [4] and [5], respectively. Common Crane observations corresponded with the species’ known migration routes from the Iberian Peninsula to northern Europe and, to a lesser extent, across the Po River Valley in northern Italy **(C)**. Base maps were provided by the University of Minnesota (https://conservancy.umn.edu/handle/11299/227302).

By reducing the barrier to entry of citizen science bird monitoring, we rapidly amassed 5.8 million high-quality bird observations across North America and Europe (Fig 1E). We then conducted 4 case studies to test whether app data could be used to replicate known patterns in avian ecology and thus serve as a reliable research resource. The case studies were selected based on the availability of results we could attempt to reproduce (e.g., song dialect mapping has been somewhat limited at broad scales because of data limitations) and to encompass a range of phenomena (e.g., simple versus complex songs and the migration of small versus large populations). First, we manually classified 1,392 White-throated Sparrow (*Zonotrichia albicollis*) song observations as ending in a doublet or triplet phrase. While [4] reported a uniform west-to-east expansion of the White-throated Sparrow’s doublet song, we found that the triplet has persisted across the western portion of the sparrow’s range and that the doublet phrase has penetrated to the far eastern edge of the range (Fig 2A). The improved detail we achieved is likely a function of sample size: The 1,392 songs we analyzed were collected in just 3 months, whereas the 1,785 songs analyzed by [4] were amassed over 70 years. Second, we manually classified 4,466 Yellowhammer (*Emberiza citrinella*) song observations as B- or X-type and found, consistent with [5], that the X-type song was dominant in Germany and southeastern England, while the B-type song was dominant in Poland and eastern Europe (Fig 2B). Third, we mapped 2,690 Brown Thrasher (*Toxostoma rufum*) observations and found that the app data accurately reflected the known seasonal and migratory ranges of this species in eastern North America (BirdLife International and Handbook of the Birds of the World [2019] Bird species distribution maps of the world. Version 2019.1. Available from: http://datazone.birdlife.org/species/requestdis). Yet, the Brown Thrasher has an estimated population of 6,100,000 [6], and its migration is essentially a massive wave of birds, and we wanted a more difficult test. Therefore, fourth, we mapped 1,700 observations of the Common Crane (*Grus grus*), which has a migratory population of approximately 250,000 that flies from the Iberian Peninsula and northern Africa to northern Europe (BirdLife International. 2020. Species factsheet: *Grus grus*). Once again, we were able to accurately map this migration, and there were even observations from a newly described flyway across the Po River plain in northern Italy used by just a few thousand individuals [7] (Fig 2C). The success of these 4 case studies suggests that the BirdNET App can generate reliable data suitable for novel inquiries in bird research. The data used in these case studies are publicly available (https://doi.org/10.5281/zenodo.6484061).

By creating an app that was easy to use and produced satisfying results, we were able to create a user community of over 2 million people globally and thus rapidly accrue tens of millions of bird observations across 6 continents that can yield accurate assessments of avian ecology. The defining characteristic of machine learning–powered nature apps like BirdNET is that digital media are converted to biological observations by the algorithm, rather than human observers. Consequently, potential biases such as species-specific classification performance will be uniform and directly measurable, as opposed to variable and uncertain, as occurs when users of unknown skill are identifying species. We have shown that eliminating the need for species identification skills and equipment removes a potentially significant barrier to citizen science participation and that the resulting data can be used to generate accurate results.

Pl@ntNet app data have been used to support conservation initiatives in Europe and Africa [8], and we believe BirdNET App data have similar potential. Expanded research applications will depend on a thorough understanding of the biases inherent in the bird observations. Variation in vocal activity across seasons and among species, user motivations, cell phone coverage, ambient noise among habitats, and species-specific classification accuracy, as well as digital divides such as the wealth needed to own a smartphone will all be manifested in BirdNET App data [3]. Nonetheless, the 4 case studies we presented reveal that app data can already provide accurate results.

A forthcoming app user data analysis portal will allow users to analyze their own observations in greater detail than the current species list and “explore your area” features, an improvement that will enable users to pursue their own research questions. Adding an optional social networking component to the app could be transformative. The ability to connect with friends could foster friendly competition that would likely increase submission rates. More importantly, pooling results across multiple users would enable community-driven projects to document local bird diversity to promote ecotourism, defend lands against resource extraction, or educational uses. An optional “point count mode” with which users could submit 3- to 5-minute continuous soundscape recordings would allow communities to document an estimated 5% to 15% of the local bird species per week even with minimal participation [9]. Moreover, these acoustic point counts would yield analytically valuable nondetections of the remaining species.

At the outset of this project, we envisioned networks of app users interacting with birds, with their own bird observations, with each other, and with researchers. In just 3 years, over 2 million users from over 100 countries have generated over 40 million submissions. Critically, these submissions are not the final product: The BirdNET App enables top-down and, soon, bottom-up research on avian ecology at continental scales. We welcome inquiries from researchers interested in using BirdNET App data (email: ccb-birdnet@cornell.edu). Raw observations are not yet publicly available due to the substantial challenges to hosting tens of millions of observations and audio files; sharing observations only (i.e., prediction scores and metadata but no audio) is simpler but prevents validation of observations and thus leaves the data vulnerable to misinterpretation. Detailed data usage guidelines that will facilitate widespread open-access data sharing are forthcoming.

The removal of barriers is the most important aspect of machine learning–powered nature apps like BirdNET. People do not need knowledgeable mentors or specialized equipment, which is often expensive, to identify the species around them, they simply need a smartphone. Challenges remain, as classification accuracy will never be perfect and gaps—both geographic and phylogenetic—in coverage will persist. However, greater accessibility means that more people have the opportunity to engage more deeply with the nonhuman world, which can potentially improve both their physical and mental health [10]. Highly accessible tools can also foster participation in other citizen science programs, such as eBird. At a societal level, making nature experiences more accessible may help make people more attuned to—and invested in—environmental conditions [11]. From a research perspective, greater accessibility means that more data can be collected more rapidly than if the citizen science participant pool were limited to wealthier and already-skilled volunteers. We hope that the BirdNET App and similar projects will enable new opportunities for avian research and conservation.

## References

[1] Affouard A, Goeau H, Bonnet P, Lombardo JC, Joly A. Pl@ntNet app in the era of deep learning. In: ICLR: International Conference on Learning Representations. 2017. p. 6.

[2] Kahl S, Wood CM, Eibl M, Klinck H. BirdNET: A deep learning solution for avian diversity monitoring. Eco Inform. 2021;61:101236. doi: 10.1016/j.ecoinf.2021.101236

[3] Andrachuk M, Marschke M, Hings C, Armitage D. Smartphone technologies supporting community-based environmental monitoring and implementation: a systematic scoping review. Biol Conserv. 2019;237:430–42.

[4] Otter KA, Mckenna A, LaZerte SE, Ramsay SM. Continent-wide Shifts in Song Dialects of White-Throated Sparrows. Curr Biol. 2020;30:1–5. doi: 10.1016/j.cub.2020.05.084 32619475

[5] Petrusková T, Diblíková L, Pipek P, Frauendorf E, Procházka P, Petrusek A. A review of the distribution of Yellowhammer (*Emberiza citrinella*) dialects in Europe reveals the lack of a clear macrogeographic pattern. J Ornithol. 2015;156(1):263–73.

[6] Rosenberg KV, Kennedy JA, Dettmers R, Ford RP, Reynolds D, Alexander JD, et al. Partners in Flight Landbird Conservation Plan: 2016 Revision for Canada and Continental United States. Partners in Flight Science Committee; 2016 p. 119.

[7] Mingozzi T, Storino P, Venuto G, Alessandria G, Arcamone E, Urso S, et al. Autumn Migration of Common Cranes *Grus grus* Through the Italian Peninsula: New Vs. Historical Flyways and Their Meteorological Correlates. Acta Ornithol. 2013 Jun;48(2):165–77.

[8] Bonnet P, Joly A, Faton JM, Brown S, Kimiti D, Deneu B, et al. How citizen scientists contribute to monitor protected areas thanks to automatic plant identification tools. Ecol Solut Evid. 2020;1(2):e12023.

[9] Wood CM, Kahl S, Chaon P, Peery MZ, Klinck H. Survey coverage, recording duration and community composition affect observed species richness in passive acoustic surveys. Methods Ecol Evol 2021 May;12(5):885–96.

[10] Bratman GN, Anderson CB, Berman MG, Cochran B, de Vries S, Flanders J, et al. Nature and mental health: An ecosystem service perspective. Sci Adv. 2019 Jul 1;5(7):eaax0903. doi: 10.1126/sciadv.aax0903 31355340 PMC6656547

[11] Soga M, Gaston KJ. Shifting baseline syndrome: causes, consequences, and implications. Front Ecol Environ. 2018;16(4):222–30.

